# Two new clades recovered at high temperatures provide novel phylogenetic and genomic insights into *Candidatus* Accumulibacter

**DOI:** 10.1093/ismeco/ycae049

**Published:** 2024-04-18

**Authors:** Xiaojing Xie, Xuhan Deng, Jinling Chen, Liping Chen, Jing Yuan, Hang Chen, Chaohai Wei, Xianghui Liu, Guanglei Qiu

**Affiliations:** School of Environment and Energy, South China University of Technology, Guangzhou 510006, China; School of Environment and Energy, South China University of Technology, Guangzhou 510006, China; School of Environment and Energy, South China University of Technology, Guangzhou 510006, China; School of Environment and Energy, South China University of Technology, Guangzhou 510006, China; School of Environment and Energy, South China University of Technology, Guangzhou 510006, China; School of Environment and Energy, South China University of Technology, Guangzhou 510006, China; School of Environment and Energy, South China University of Technology, Guangzhou 510006, China; Guangdong Provincial Key Laboratory of Solid Wastes Pollution Control and Recycling, Guangzhou 510006, China; Singapore Centre for Environmental Life Sciences Engineering, Nanyang Technological University, Singapore 637551, Singapore; School of Environment and Energy, South China University of Technology, Guangzhou 510006, China; Guangdong Provincial Key Laboratory of Solid Wastes Pollution Control and Recycling, Guangzhou 510006, China; Singapore Centre for Environmental Life Sciences Engineering, Nanyang Technological University, Singapore 637551, Singapore; The Key Lab of Pollution Control and Ecosystem Restoration in Industry Clusters, Ministry of Education, Guangzhou 510006, China

**Keywords:** Candidatus Accumulibacter, enhanced biological phosphorus removal (EBPR), machine learning, comparative genomics, heat shock protein

## Abstract

*Candidatus* Accumulibacter, a key genus of polyphosphate-accumulating organisms, plays key roles in lab- and full-scale enhanced biological phosphorus removal (EBPR) systems. A total of 10 high-quality *Ca*. Accumulibacter genomes were recovered from EBPR systems operated at high temperatures, providing significantly updated phylogenetic and genomic insights into the *Ca*. Accumulibacter lineage. Among these genomes, clade IIF members SCELSE-3, SCELSE-4, and SCELSE-6 represent the to-date known genomes encoding a complete denitrification pathway, suggesting that *Ca*. Accumulibacter alone could achieve complete denitrification. Clade IIC members SSA1, SCUT-1, SCELCE-2, and SCELSE-8 lack the entire set of denitrifying genes, representing to-date known non-denitrifying *Ca*. Accumulibacter. A pan-genomic analysis with other *Ca*. Accumulibacter members suggested that all *Ca*. Accumulibacter likely has the potential to use dicarboxylic amino acids. *Ca*. Accumulibacter aalborgensis AALB and *Ca*. Accumulibacter affinis BAT3C720 seemed to be the only two members capable of using glucose for EBPR. A heat shock protein Hsp20 encoding gene was found exclusively in genomes recovered at high temperatures, which was absent in clades IA, IC, IG, IIA, IIB, IID, IIG, and II-I members. High transcription of this gene in clade IIC members SCUT-2 and SCUT-3 suggested its role in surviving high temperatures for *Ca*. Accumulibacter. Ambiguous clade identity was observed for newly recovered genomes (SCELSE-9 and SCELSE-10). Five machine learning models were developed using orthogroups as input features. Prediction results suggested that they belong to a new clade (IIK). The phylogeny of *Ca*. Accumulibacter was re-evaluated based on the laterally derived polyphosphokinase 2 gene, showing improved resolution in differentiating different clades.

## Introduction

Phosphorus (P) is a nonrenewable resource and a key water pollutant [1]. Enhanced biological phosphorus removal (EBPR) is an economical and efficient process widely applied in municipal wastewater treatment plants (WWTPs). It is mediated by polyphosphate-accumulating organisms (PAOs), which have an extraordinary ability to utilize the intracellularly stored polyphosphate (poly-P) as an energy source to take up simple organic compounds like volatile fatty acids (VFAs) under anaerobic conditions [2]. The organic compounds are stored in the forms of polymers, such as polyhydroxyalkanoic acids (PHAs) and/or glycogen [3]. In the aerobic phase, PAOs oxidize the stored polymers, producing ATP and taking up phosphate to synthesize poly-P [4, 5]. Via an anaerobic–aerobic cycle, phosphate in the wastewater is transferred into PAO cells in the activated sludge, achieving P removal [6, 7].

*Candidatus* Accumulibacter is a key group of PAOs predominating lab- and full-scale wastewater treatment systems [8–10]. The polyphosphokinase 1- encoding gene (*ppk*1) serves as a high-resolution biomarker that classifies them into two major types, i.e. type I and type II [11, 12], and several clades (IA-IH and IIA-IIJ) [13–16].

The genomic makeup of *Ca*. Accumulibacter members were intensively studied over the last years [17–19], benefiting an increasingly comprehensive and holistic understanding of this group of bacteria [9, 20–22]. However, there are still controversies in a few of their key metabolic potentials in terms of nitrogen metabolism, carbon utilization, temperature adaptability, etc. A key contradictory was their ability to couple P uptake with nitrate/nitrite reduction. Combined fluorescence in situ hybridization (FISH) and physiochemical (in the form of batch tests) analyses attributed the complete denitrification ability to clade I *Ca*. Accumulibacter, while clade II was thought to rely solely on oxygen and nitrite [23–25]. Kim et al. demonstrated that clades IA, IIA, IIC, and IIF members were not able to use nitrate, contrasting experiments performed by Zeng et al. and Flowers et al. which supported denitrification by clades IIC and IA members [25, 26]. Additionally, Rubio-Rincón et al. obtained an enrichment culture of a clade IC member *Ca*. Accumulibacter delftnsis which was not capable of using nitrate as the electron acceptor [19]. Camejo et al. demonstrated that another IC member *Ca*. Accumulibacter meliphilus UWLDOIC encoded a complete pathway for respiratory denitrification [22, 27], suggesting that a high-level eco-physiological diversity may have been preserved in the sub-clade level of *Ca*. Accumulibacter lineage. However, a systematic examination of 36 *Ca*. Accumulibacter genomes showed that no member encoded a complete denitrifying pathway, and the gene distributions did not support the hypothesized physiological differences between and within clade I and clade II [10]. Since *Ca*. Accumulibacter members encoding a complete denitrification pathway have not been found, it is still unknown whether *Ca*. Accumulibacter can perform complete denitrification individually or via community-level cooperation. Additionally, novel carbon source utilization capability was observed for *Ca*. Accumulibacter enrichment cultures [28, 29]. For instance, *Ca*. Accumulibacter was previously believed incapable of using glucose as a carbon source for EBPR. However, in a recent study, an enrichment culture of clade IIA members was shown to directly use glucose [29]. Additionally, a clade IIF member *Ca*. Accumulibacter similis SCELSE-1 was shown to be able to take up a range of amino acids anaerobically [28]. These observations challenged the previous understanding, where *Ca*. Accumulibacter- and *Tetrasphaera*-related PAOs occupied distinct ecological niches in EBPR systems owing to their distinct carbon source preferences [7, 30]. It is not clear if these carbon utilization capabilities are widely preserved within the genus, or clade/species specific. Additionally, the feasibility and stability of EBPR and the underlying microbial ecological basis at high temperatures (>25°C) have been another topic receiving widespread attention, especially, in the context of global warming. 16S rRNA gene-based phylogeny analysis suggested that *Ca*. Accumulibacter enriched/occurred at elevated temperatures (30°C and 35°C) are commonly detected in temperate environments, suggesting that high-temperature EBPR might not require a highly specialized *Ca*. Accumulibacter community [31]. However, the observation does not exclude the possibility that certain clades/species may have exceptional abilities to survive high temperatures. Based on previous studies, it seems that, in high-temperature EBPR systems, clade I members were scarcely detected. Clade IIF and IIC were commonly observed as predominant community members, with occasional detection of clade IIA, IID, and IIB members [32–34]. The high occurrence of clade IIF and IIC members may imply their potential advantages in surviving a warmer environment. However, the genetic basis for this potential was yet to be analyzed owing to the absence of high-quality genomes or limited genome integrity recovered at high-temperature systems.

For the understanding of the intra- and inter-lineage metabolic diversities of a group of microorganisms, a robust and reliable phylogeny and clade classification is a prerequisite. However, a few members (such as those belonging to *Ca*. Accumulibacter aalborgensis) do not seem to adhere to the *ppk*-1 base population structure, but fell within the *Ca*. Accumulibacter lineage based on the single-copy core gene phylogeny [35, 36]. Clades classification using *ppk*1 may not sufficiently/correctly represent the evolutionary relationships among different *Ca*. Accumulibacter members. To overcome the disadvantage of single-gene classification, modern evolutionary studies employ multiple genes and the whole-genome data for more robust and accurate clade identification [37]. Machine learning is particularly strong in analyzing complex data and identifying trends and patterns that can easily be overlooked by traditional measures, showing promising power in classifying bacteria [38–40]. The combination of machine learning and whole-genome data has the potential to achieve a more rapid and accurate classification. It is necessary and interesting to re-analyze the phylogenetic structure of different *Ca*. Accumulibacter members via a combination of machine learning and pangenome in addition to the traditional *ppk*1-base clade classification. Additionally, compared with the ancestral gene *ppk*1, *ppk*2 is a laterally derived gene that was obtained by *Ca*. Accumulibacter at their last common ancestor (LCA), playing a key role in the emergence of the polyphosphate accumulating feature of *Ca*. Accumulibacter [41]. It has the potential to be a more pertinent gene maker to reflect the genetic variation of different lineage members during evolution and speciation.

In this study, 10 high-quality *Ca*. Accumulibacter genomes were recovered from lab-scale reactors operated at high temperatures. Two of them belong to clade IIH. Another two genomes were distinctly branched from other clade members. Machine-learning-guided genome-centric pangemomics, and phylogenetic analysis based on the *ppk*2 gene were used to analyze and resolve the clade identity of these two genomes, and to re-analyze the phylogeny of the *Ca*. Accumulibacter genus. Using these 10 genomes together with all available high-quality genomes in the GeneBank database, a systematic pangenomic analysis was performed. The genomic features and metabolic characteristics of different clade members were compared and analyzed together with the exploration of their genetic basis for surviving high temperatures. These novel high-quality genomes, including representative genomes of two novel clades, provide important insights into the nitrogen and carbon metabolisms, and heat stress adaption mechanisms of *Ca*. Accumulibacter, resulting in an improved understanding of genomic differences within and among clades, especially those with important ecological significance.

## Material and methods

### Sequencing batch reactor operation

Two sequencing batch reactors (SBRs) (with a working volume of 1.59 L, operated at 30°C and 35°C, respectively) were inoculated with activated sludge from a WWTP in Singapore [32]. The SBRs were operated with 6 h cycles. A total of 12 activated sludge samples were collected from these SBRs on Days 14, 56, 91, 201, 280, and 301 for metagenomic analysis. The third SBR (with an effective volume of 4.5 L) was inoculated with activated sludge from a WWTP in Guangzhou, China, for the enrichment of *Ca*. Accumulibacter at 25°C. Activated sludge samples were collected on Day783 for metagenomic analysis [20] (More details on the operational conditions of these SBRs are documented in the Supplementary Materials S1–S3). An anaerobic–aerobic full-cycle study was performed to investigate the gene transcription activities of the enrichment culture in the third SBR. Activated sludge samples were collected just before the start of an SBR cycle (0 min), and at 5 min (anaerobic phase), 30 min (anaerobic phase), 105 min (aerobic phase), and 120 min (aerobic phase) of the SBR cycle. The samples were snap-frozen in liquid N_2_ and stored at −80°C before the ribonucleic acid (RNA) extraction for metatranscriptomic analysis. More details on the metagenomic and metatranscriptomic analyses are documented in the Supplementary Materials (S4 and S5). Raw reads and metagenome-assembled genomes (MAGs) obtained were submitted to NCBI under BioProject No. PRJNA807832 and PRJNA771771.

### Data acquisition and evaluation

The completeness and contamination of the *Ca*. Accumulibacter genomes were evaluated using CheckM. A total of 45 genomes with completeness of >95% and contamination <6% were used for analyses [31, 42, 43], including 13 high-quality genomes recovered from our EBPR reactors [20, 41, 44, 45] and 32 genomes obtained from the NCBI database [32, 44]. The average nucleotide identity (ANI) value between each two genomes was calculated using FastANI [46]. The GenBank assembly accession, corresponding species names, and additional details about the qualities of these genomes are in the Supplementary Spreadsheet S1.

### Orthologue analyses

Orthologous gene clustering is necessary for the analysis of the metabolic differences within and among clades and for reliable phylogenic analysis. For orthologous gene cluster identification, all vs all BLAST of each *Ca*. Accumulibacter genome was performed using Orthofinder 2.5.4 with parameters - evalue 1e-5, -seg yes, -soft_masking true, -use_sw_tback. The obtained results were then filtered to a query coverage of 75% and an identity of 70% [47]. Orthologous gene clusters were identified using MCL version 14–137 with an inflation value of 1.1 [48] (the detailed results of the orthologue analyses are documented in the Supplementary Spreadsheet S2). An orthogroup is considered to be present in a clade if it appears in >60% of all clade members. To study the genomic makeup characteristics of the two novel clades (clade IIH and IIK), core genes (genes present in all clades), clade-specific genes (genes present in all members of a specific clade but not in other *Ca*. Accumulibacter genomes), and flexible genes (genes present in more than one but not all clades) were identified. Using the STAG algorithm, rootless trees were constructed using all genes instead of single-copy genes [47]. The STRIDE algorithm is used to infer rooted trees from the rootless trees. The *ppk*1 and *ppk*2 gene sequences were obtained from the NCBI database. Phylogenetic analyses were performed using iqtree2. Trees were constructed based on the maximum likelihood method with parameter -B 1000 -T30, using the best-fitting model GTR + F + I + G4 and TPM3u + F + I + G4 for *ppk*1 and *ppk*2 [49]. Landscaping of the phylogenetic trees was done using Chiplot [50].

### Metabolic function analysis

To investigate the metabolic pathways, genomes were annotated based on KEGG [51]. The following nomenclature was used to describe the presence and completeness of pathways. “C” for pathways that were found to be fully intact. “I” for incomplete pathways. The number following the letter “I” indicates the number of existing gaps. In cases where no related genes or information about a pathway were identified in a genome, it was labeled as “-” to signify the absence of a particular pathway (Supplementary Spreadsheet S1). Additional analysis and validation (e.g. for the presence of the full glucose metabolic gene cluster) were performed by annotating the genomes using the RAST annotation server [52, 53] to confirm the presence/absence of key genes to ensure consistency and reliability of the results.

### Machine learning analysis

To comprehensively analyze and distinguish the key genetic characteristics of each clade, as well as to determine the clade identity of SCELSE-9 and SCELSE-10, a novel clade classification method was developed based on machine learning.

A total of 39 high-quality genomes with known clade identities were used to train the machine-learning models. A comparative genomic analysis was performed to identify orthogroups. The orthogroup table was obtained by setting a similarity threshold for clustering the genes. Orthogroups were used as input features [47]. Given the excessive number of features (orthogroups), a feature selection process was implemented. Specifically, homologous gene clusters with low variation among clades were excluded, helping to reduce the dimensionality of the dataset and focusing on the most informative and relevant features. To address the potential class imbalance issue in the dataset, an upward sampling method (to a number of 9) was employed, ensuring that each clade was adequately represented during model construction. The obtained features are trained with the classifier (70% for training, and the remaining 30% for testing). Additionally, 5-fold cross-validation was performed, which involves dividing the data into five equal parts: one part for testing and the remaining four parts for training in each experiment for model evaluation. This cross-validation process helped in evaluating the robustness and generalization performances of the models.

Five different models, including gradient boosting machine (GBM) [54], random forest (RF) [55], support vector machine (svmRadial, svmLinear) (SVMR, SVML) [56], and LogitBoost (LB) [57], were developed and evaluated for their prediction accuracy. Each model underwent 10 repetitions for accuracy testing, allowing for a comprehensive assessment of the algorithm’s performance. All information for machine learning models including input data and output results are provided in the Supplementary Spreadsheet S2.

## Results and discussion

### Metagenome assembly

**Figure 1:**
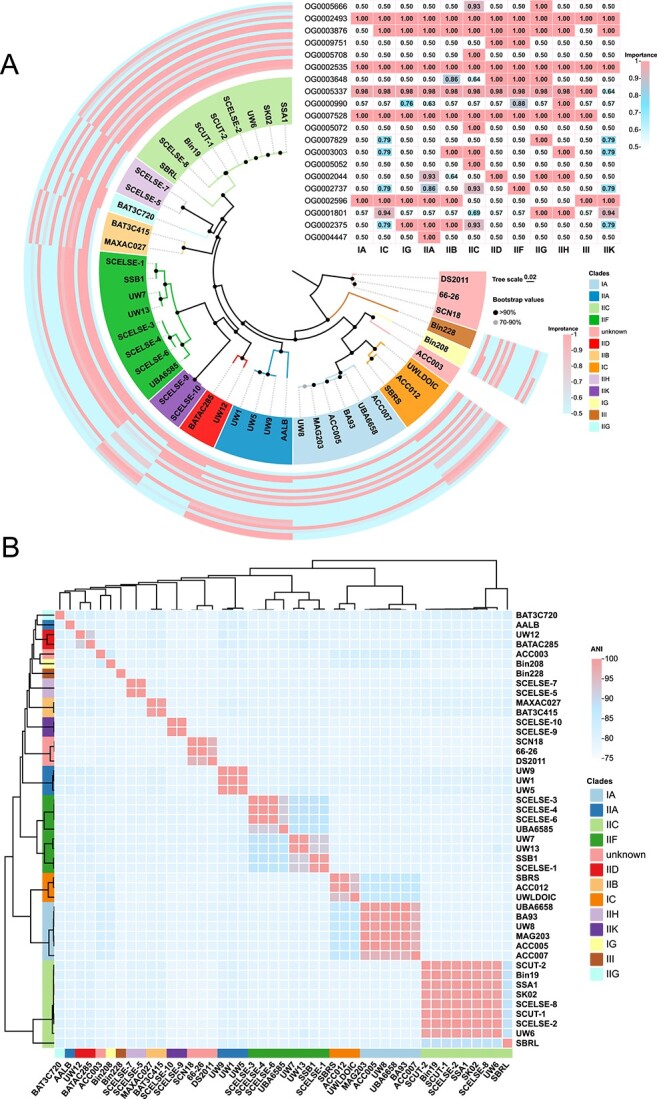
(A) A whole-genome phylogenetic tree of 45 *Ca*. Accumulibacter was built using Orthofinder [47]; using the STAG algorithm, rootless trees were constructed using all genes instead of single-copy genes [47]; the STRIDE algorithm is used to infer rooted trees from the rootless trees; the gray dots suggest bootstrap values between 70% and 90%; the black dots denote values >90%; for NCBI GenBank genome accession numbers and leaf names, see Supplementary Spreedsheet S1; five machine learning models were developed to reanalyze the phylogeny of the *Ca*. Accumulibacter genus; the heatmap represents the 20 most important features for clade classification extracted from the LB model; the values within the heatmap showed the importance (ranging from 0 to 1, with 1 being the highest importance value) of each feature to different clades; (B) an ANI analysis of *Ca*. Accumulibacter genomes with FastANI [46].

From three lab-scale SBRs, 10 high-quality (completeness >95% and contamination <6%) *Ca*. Accumulibacter genomes were recovered [32, 44]. All of them are clade II members, including three clade IIC members, three clade IIF members, and two clade IIH members. Two genomes were distinctly branched from other known clade members, which was later shown to represent a novel clade (IIK) in this study. This is the first time to report the successful recovery of clade IIH and IIK member genomes (the representing species are named as *Ca*. Accumulibacter tropicus and *Ca*. Accumulibacter torridus, respectively). Description of “*Candidatus* Accumulibacter torridus” sp. nov.: “*Candidatus* Accumulibacter torridus,” (*tor'ri.dus. L. part, torridus,* burned; referring to the fact that the species was recovered from a warm environment). Description of “*Candidatus* Accumulibacter tropicus” sp. nov.: “*Candidatus* Accumulibacter tropicus,” (*tro'pi.cus. L. masc. adj. tropicus* tropical, indicating this species have adapted to warm conditions). A phylogenic tree of these *Ca*. Accumulibacter genomes was shown in Fig. 1A.

### Classification of *Ca*. Accumulibacter torridus SCELSE-9 and SCELSE-10

The genetic relationships among 45 *Ca*. Accumulibacter genomes were further elucidated via an ANI analysis [58] (Fig. 1B). ANI values of around 95%–96% are typically used as a criterion to delimit different species [59]. In our previous phylogenetic analysis, *Ca*. Accumulibacter torridus SCELSE-9 and SCELSE-10 formed a supercluster with clade IIF members [20], whereas, with low identity values (<79%) (Fig. 1B). Additionally, the *ppk*1-base similarity of SCELSE-9 and SCELSE-10 with clade IIF members was 84%, significantly lower than those (89% at the least) among IIF members. In terms of gene clusters, 1886 orthogroups were shared among clade IIF members and SCELSE-9 and SCELSE-10, whereas more are differential ones (2453 orthogroups, 1118 are clade IIF member specific; 1335 are SCELSE-9 and SCELSE-10 specific), suggesting their major differences.

To further determine the clade identity of SCELSE-9 and SCELSE-10 and to re-assess the phylogenetic relationships among different *Ca*. Accumulibacter members, five machine learning models were developed. 100% prediction accuracy was achieved for clade identity prediction of all genomes, except that inconsistent results were obtained for SCELSE-9 and SCELSE-10. GBM showed that they belong to clade IIA; SVML showed that they are IIF members; and LB, SVMR, and RF suggested that they affiliate to clade II-I.

This discrepancy further underscored the need for a novel clade designation. The branch, which SCELSE-9 and SCELSE-10 are affiliated to, was thus proposed as clade IIK. The species represented by SCELSE-9 and SCELSE-10 is named as *Ca*. Accumulibacter torridus given their prevalence in the bioreactors operated at elevated temperature (35°C). The LB model identified a few key features distinguishing different clade members. Features with the top 20 importance were extracted (Supplementary Spreadsheet S2 and Fig. 1A). Eight of them were obtained via horizontal gene transfer (HGT), indicating the contribution of HGT to the genetic differences among clades (Supplementary Spreadsheet S2).

A combination of ANI analysis and machine learning led to clearer allocations of clades, and contributed to an improved understanding of the evolution relationships among different members.

### General features of the *Ca*. Accumulibacter genomes

**Figure 2:**
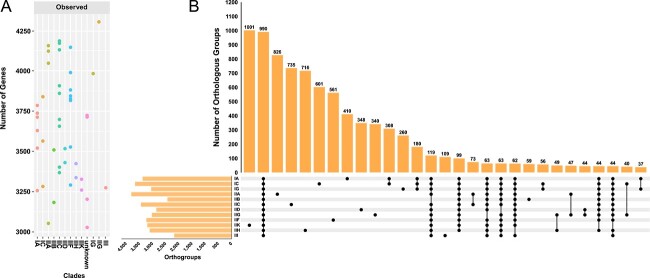
(A) The total number of genes observed in each clade member genomes; (B) an upset plot representing the intersections of groups of orthologous gene clusters among each clade; the longitudinal bar chart represents the number of orthologous gene clusters; the dot plot represents the genes in which the groups intersect; the lateral bar chart shows the numbers of orthologous genes encoded by each clade.

The genome sizes of *Ca*. Accumulibacter members ranged from 3.7 to 5.7 MB, with an average value of 4.6 MB. There was not a clear relationship between genome sizes and clade identities. Previous studies suggested that genome size is influenced by ecological niches and nutrition strategies [60]. The forces driving the evolution in the *Ca*. Accumulibacter genome size remain unknown. All members showed a relatively stable G + C content range (61%–66.5%, averaged at 63.5%), showing an overall genetic stability within the genus. Orthofinder identified 16 524 homologous gene clusters. Clade IIA had the highest number (3809). Clade II-I had the least (2180) (Fig. 2B). There was no significant correlation between the number of genes and clade identities (Fig. 2A). Detailed analyses of clade IIH and IIK revealed important information about their genetic characteristics. The clade IIH member *Ca*. Accumulibacter tropicus SCELSE-7 had the highest numbers of specific genes in the inorganic ion transport and metabolism pathway [35] and the replication, recombination, and repair pathway [53], indicating that the organism experienced major evolutionary pressures in developing efficient ion transport and DNA maintenance. The clade IIK member *Ca*. Accumulibacter torridus SCELSE-10 showed the greatest numbers of specific genes in cell wall/membrane/envelope biogenesis [61], suggesting its potentially strong reliance in extreme environments. This aligned with the high-temperature environment in which SCELSE-10 was recovered.

### Polyphosphokinase 2 gene (ppk2) phylogeny for clade identification

**Figure 3:**
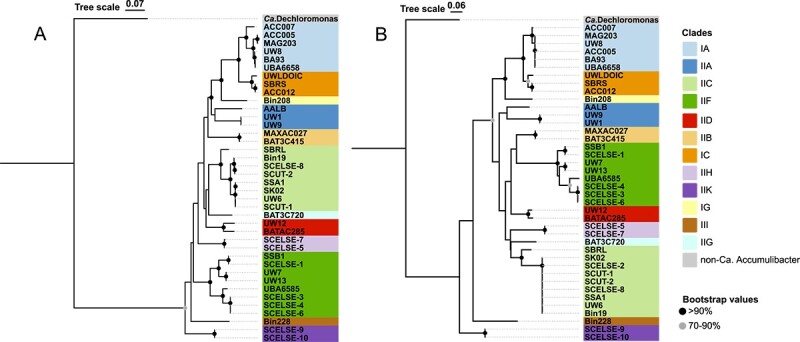
(A) A phylogenetic tree based on *ppk*1; (B) a phylogenetic tree based on *ppk*2; the *ppk*1 and *ppk*2 sequences were obtained from the NCBI database; phylogenetic analyses of these two genes were performed using iqtree2; the maximum likelihood trees were constructed with parameter -B 1000 -T30, using the best-fitting model GTR + F + I + G4 and TPM3u + F + I + G4 for *ppk*1 and *ppk*2; bootstrap values obtained from 1000 re-samplings are indicated at the node of each branch; the gray dots suggest bootstrap values between 70% and 90%; the black dots denote values >90%. *Ca*. Dechloromonas phosphoritropha FLASV138 (MiDAS database) (GCA_016722705.1) [62] was used as the outgroup; the scale bar represents phylogenetic distance of nucleotide substitutions per site.

In *Ca*. Accumulibacter genomes, there are multiple copies of polyphosphokinase 2 (*ppk*2) genes. One of them was obtained via HGT at their LCA, and was highly transcribed in SCUT-2 in an EBPR cycle [41]. Our previous study suggested that *ppk*2 might be a key gene contribution to the polyphosphate-accumulating capability of *Ca*. Accumulibacter [41]. It has the potential to be an alternative and supplementary gene to characterize and differentiate different clades. A phylogenetic analysis was performed on *ppk*2 (Fig. 3). Members from the same clades grouped together, showing the effectiveness of *ppk*2 as a genetic marker. Nevertheless, compared with the *ppk*1 tree (Fig. 3A), the *ppk*2 tree (Fig. 3B) seemed to show improved resolution in clade identification, since it exhibited generally greater distances among clades with overall reduced intra-clade distances. A combined use of the *ppk*1 and *ppk*2 phylogenies may benefit an increasingly robust and reliable classification of *Ca*. Accumulibacter clades without losing the intra-clade phylogenetic resolutions.

### Metabolism analysis

#### *Ca*. Accumulibacter core metabolism

**Figure 4:**
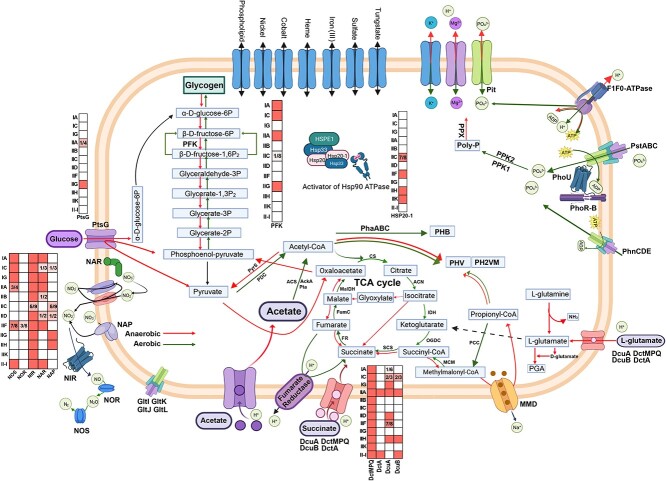
Reconstruction of pan-metabolic pathways of *Ca*. Accumulibacter (created via BioRender.com); heatmaps beside key pathways show the presence of key genes in each clade; the filled grid without numbers in the heatmap means that the corresponding gene occurs in all members of this clade; the blank grid without numbers indicates that the corresponding gene was absent in all members of a clade;the numbers in the grid in the format of “x/y” mean that there were a total number of “y” genomes in this clade, of which “x”genomes encoded the corresponding gene.

The reconstruction of core metabolic pathways provided novel insights into their metabolic properties (Fig. 4). Phosphate metabolism-related genes are highly conserved, including those involved in phosphate uptake, storage, and utilization. The low-affinity (*pit*) and high-affinity phosphate transporter (*pst*) genes are essential for phosphate release and uptake. Polyphosphate kinase 1 and 2 genes (*ppk*1 & *ppk*2) and exopolyphosphatase genes (*ppx*) are keys for polyphosphate synthesis and hydrolysis, repsectively. The phosphate regulon (Pho) is a global regulatory mechanism to manage intracellular inorganic phosphate concentrations, which requires seven proteins in signal transduction, including PhoR, PhoB, Pst (PstS, PstA, PstB, and PstC), and PhoU. Adaptation to phosphate limitation is typically mediated by the two-component signal transduction system PhoR-B [63]. PhoU protein regulates Pst-mediated phosphate uptake and PhoR-B-mediated phosphate restriction response [64]. PhoR-B is conserved in all *Ca*. Accumulibacter genomes. Apart from the PstSCAB, genes encoding a PhnCDE system were observed in clades IA, IC, IG, IIA, IIC, and IID members. PhnCDE was shown to function as a high-affinity phosphate transporter in *Mycobacterium smegmatis* [65].

**Figure 5:**
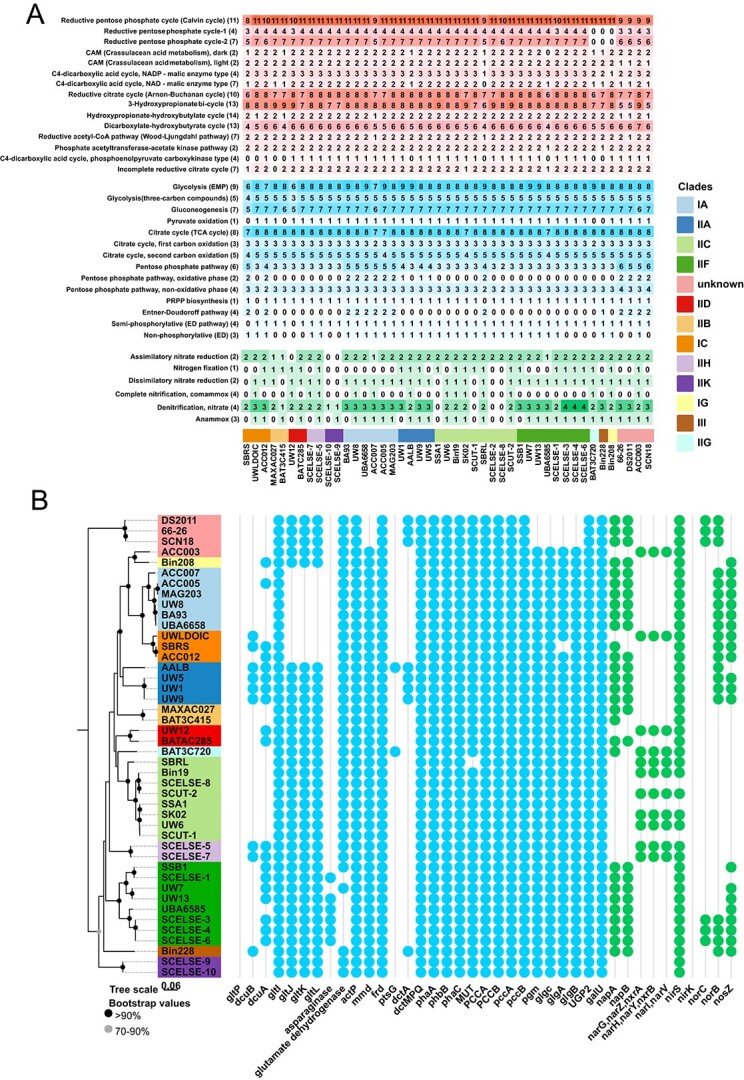
(A) Carbon and nitrogen metabolic modules in the representative genomes of each clade; the numbers following the module names indicate the number of genes in each module; the numbers in the heatmap indicate the number of genes observed in each genome; (B) the presence or absence of key genes in carbon and nitrogen metabolism; differential genes in different *Ca*. Accumulibacter members are focused; for the full list of gene names and associated KO numbers, see Supplementary Spreadsheet S1.

Carbon fixation pathway, i.e. the Calvin cycle, is highly conserved in the *Ca*. Accumulibacter genus, indicating their universal adaption to resource-limited environments. Moreover, PHA metabolism-related genes (such as *pha*A, *pha*B, *pha*C, *pcc*B, and *pcc*A, Fig. 5) are widely preserved in all *Ca*. Accumulibacter genomes. Except for unknown clade members (DS2011, 66-26, SCN18), glycogen metabolism-related genes were also highly conserved. Based on the KEGG/RAST annotation and comparison analyses, a large number of key genes for the glycogen metabolism pathway were missing in unknown clade members (DS2011, 66-26, SCN18). Only genes encoding the phosphomannomutase/phosphoglucomutase (pmm-pgm, K15778) are observed. Since they are MAGs recovered from nontraditional phosphorus removal environments (e.g. a tap of groundwater-sourced drinking water system) with an unknown clade identity and distinct phylogenetic relationships with other well-characterized *Ca*. Accumulibacter members (Fig. 3), the meanings of the incomplete glycogen metabolism pathway in those unknown clade members are not clear.

#### Carbon metabolisms

**Figure 6:**
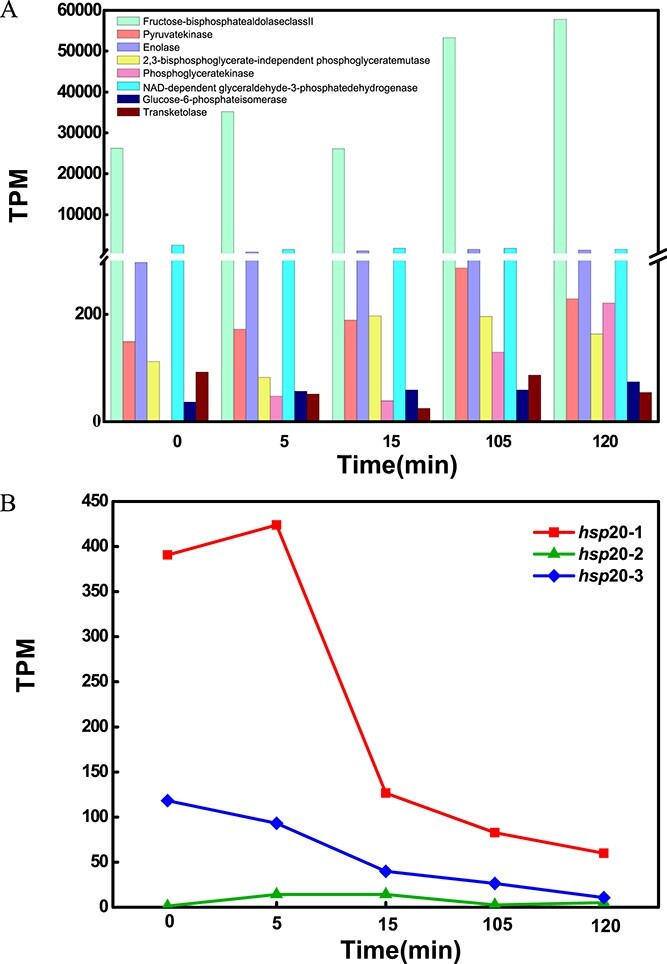
(A) The transcription patterns of the genes related to glycolysis in clade IIC member SCUT-2 during an anaerobic–aerobic full-cycle; (B) the transcription of Hsp20 family genes in clade IIC member SCUT-2; a transit of the operation conditions from anaerobic to aerobic occurred at 80 min.

In the central carbon metabolism, all clades encoded the complete citrate cycle (TCA cycle), gluconeogenesis, and glycolysis (Fig. 5). Glycolysis is an essential process for the generation of ATP and NADH for anaerobic carbon uptake and storage by *Ca*. Accumulibacter [61]. Glycolysis may be achieved via two pathways, i.e. the Embden–Meyerhof–Parnas (EMP) pathway and the Entner–Doudoroff (ED) pathway [66]. Most bacteria and eukaryotes prefer the EMP pathway, since it enables the production of more ATP [67, 68]. It was unexpected that, apart from clade IA members BA93, UBA6658, and ACC005, IIA members UW1 and AALB, IIC member SBRL, IIG member BAT3C720, and IIF members UW7 and UW13, all other genomes lack the 6-phosphofructokinase A gene (*pfk*A, K00850) in the EMP pathway. Clade IA and IIA members (UW8, MAG203, ACC007, UW5, and UW9) encoded a homolog of the *pfk*A (OG0002868), which was derived horizontally from *Candidatus* Competibacteracea. Given the highly conserved glycolysis capability of *Ca*. Accumulibacter, we hypothesized that there might be novel *pfk*A genes in IIC IIB, IID, IIH, and IIK members. To validate this hypothesis, the transcriptome of the clade IIC member SCUT-2 was analyzed (Fig. 6A), results showed that all other genes in the EMP pathway were highly transcribed which was consistent with the transcriptome of UW1 (having *pfk*A) [17], suggesting that there are uncharacterized *pfk*A genes in the SCUT-2 genome (and probably in IG, IIB, IID, IIH, IIK, II-I members as well). Previous research suggested that, in clade I, the amount of reducing equivalents (NADH) generated via glycolysis was typically insufficient to reduce and condense acetate [69]. A part of the acetate was routed to the TCA cycle for the generation of reducing equivalents [2, 69]. In contrast, glycogen-accumulating organisms (GAOs) (e.g. *Ca*. Competibacter) solely rely on glycolysis to generate ATP and NADH for acetate storage; excessive reducing power was consumed via the succinate–propionate pathway [70]. *Ca*. Accumulibacter clade II (mainly IIC and IID) was shown to use a mixed clade I and GAO metabolism [69, 71]. The differences in the *pfk*A gene between those clade I and II members might have resulted in their different behaviors in reducing power generation for acetate storage.

Different from the EMP pathway, a large number of genes in the ED pathway are missing in *Ca*. Accumulibacter genomes, indicating their inability to use this pathway. Glucose-6-phosphate dehydrogenase (catalyzing the first step of the ED pathway) activity tests of the cell-free extract from an enrichment culture of clade I *Ca*. Accumulibacter also suggested that the ED pathway was not working during anaerobic acetate uptake [69]. Additionally, only 66-26 and SCN18 (unknown clade) encoded a complete pentose phosphate pathway (M00004, M00006, M00007), implying an extra choice for glycolysis by these members.

Glucose utilization has been a recurring topic in EBPR research [29,72]. Previous studies suggested the inability of *Ca*. Accumulibacter to directly take up glucose under anaerobic conditions [72, 73]. However, in a lab-scale SBR, Agustina et al. [29] showed physicochemical and proteomic evidence of an enrichment culture of *Ca*. Accumulibacter directly utilization of glucose. This study hypothesized that glucose is stored mainly as glycogen and partly as PHA by *Ca*. Accumulibacter [29]. Metabolically and genetically, although the *Ca*. Accumulibacter genomes had relatively complete genes for glycolysis and glycogen synthesis (absence only in SCN18, DS2011, and 66-26) as well as PHA synthesis (present in all genomes), the inability of a majority of them to use glucose was a result of the lack of genes specific for glucose uptake. Of the 45 genomes, apart from clade IIA member AALB (i.e. *Ca*. Accumulibacter aalborgensis) and IIG member BAT3C720, all genomes lack a gene encoding the glucose-phosphoenolpyruvate phosphotransferase subunit G (*pts*G, K02799) based on KEGG and RAST. i.e. AALB and BAT3C720 encoded all necessary genes for glucose uptake and utilization (Fig. 5B), implying their potential to directly utilize glucose for EBPR. The genetic composition results coincided that species/strains related to *Ca*. Accumulibacter aalborgensis were key community members of the *Ca*. Accumulibacter enrichment culture in the study of Agustina et al. [29], suggesting that the observed anaerobic glucose utilization ability was most likely attributed to the presence of species/strains related to *Ca*. Accumulibacter aalborgensis. Based on the NCBI best match result, the *pst*G encoded by AALB and BAT3C720 were horizontally transferred from *Chromatiales* members, which may have rendered their unique capacity in anaerobic glucose uptake and utilization.

The ability to utilize amino acids as carbon sources was previously considered as a distinguishing physiological feature of *Tetrasphaera* which occupy distinct ecophysiological niches in EBPR systems from *Ca*. Accumulibacter [28, 73–77]. Recently, Clade IIF member SCELSE-1 was shown to use amino acids for EBPR [28]. In the pan *Ca*. Accumulibacter genome, C4-dicarboxylate transport encoding genes are classified into three groups: *dcu*AB (K07791, K07792), *dct*A (K11103), and *dct*MPQ (K11690, K11688, K11689) [78]. DutMPQ uses cation gradients to drive the import of C4-dicarboxylates and amino acids against their concentration gradient [79]. All *Ca*. Accumulibacter genomes encode the *dct*MPQ genes, suggesting their highly conserved potential to take up dicarboxylic amino acids (e.g. aspartate and glutamate) as observed for clade IIF member SCELSE-1. In *Escherichia coli*, *dct*A catalyzes the uptake of C4-dicarboxylates (succinate, fumarate, and malate) and aspartate [80], which occurred in 10 genomes (Unknown: 66-26, DS2011and SCN18, IIA: AALB, UW1, UW5 and UW9, II-I: Bin228, and IIH: SCELSE-5 and SCELSE-7). *dcu*AB is required for growth by fumarate respiration and succinate antiport, and catalyzes C4-dicarboxylates uptake and efflux. The genes encoding the C4-dicarboxylate transport protein (*dcu*A and *dcu*B, their encoding protein operated preferentially as exchange or uptake carriers) were horizontally transferred from Burkholderiales. *dcu*B (the transcription of which was shown to be fully repressed under aerobic conditions) [78, 81] occurred in clades IC, IIA, IID, IIH, and II-I genomes. *dcu*A was observed in clades IC, IG, IIA, IID, IIF, and IIH genomes. Clade IIH and IIA members encoded both *dcu*A and *dcu*B, possibly leading to their enhanced abilities in amino acid uptake.

In addition, all genomes were deficient in genes encoding the NADH-dependent isocitrate dehydrogenase (IDH3, K00030), which may result in their similar metabolic characteristics as *Ca*. Accumulibacter similis SCELSE-1, where a majority of glutamate was unable to be channeled into the TCA cycle for PHA synthesis [28].

#### Nitrogen metabolism

Major differences occurred in their nitrogen metabolism potentials (Supplementary Spreadsheet S1). Except for clade IIB, IIK, and IID members, *Ca*. Accumulibacter encoded a complete assimilative nitrate reduction pathway, showing their potential to anabolize nitrate. Clade IIK members were found to have the least number of genes (The cytochrome cd1-containing nitrite reductase gene, *nir*S, K15864, only, Fig. 5) related to nitrogen metabolism, showing their major differences from IIF members. Complete nitrogen fixation pathway occurred in half of the genomes with no obvious relation to clade identity. These nitrogen-fixation genes are ancestral. A few clade members lost some of these genes, suggesting that nitrogen-deficiency was not a universal pressure driving the evolution of *Ca*. Accumulibacter.

Denitrification is the key process in wastewater treatment involving NO_3_^—^N reduction to NO_2_^—^N and gaseous nitrogen species (NO, N_2_O, and N_2_) [82]. There have been inconsistent findings as to whether or not *Ca*. Accumulibacter could perform complete denitrification [25, 26, 83]. For example, Saad et al. showed that significant P uptake was not observed for a highly enriched clade IC member (with a biovolume>99%, based on FISH) before or after acclimatization to nitrate, indicating their incapability to perform anoxic P uptake with nitrate [19], whereas, in batch tests operated under cyclic anaerobic and microaerobic conditions, Camejo et al. demonstrated that respiratory denitrification-related genes of clade IC member* Ca*. Accumulibacter phosphatis (UWLDOIC) were highly transcribed, suggesting that this IC member was able to perform complete hypoxic denitrification coupling P uptake [24]. The KEGG annotation results indicate that only clade IIF members SCELSE-3, SCELSE-4, and SCELSE-6, which were recovered from our high-temperature SBRs, encoded the complete denitrification pathway (i.e. form nitrate to N_2_), whereas they encoded the periplasmic nitrate reduction pathway (NapAB, K02567, K02568) instead of the respiratory nitrate reduction pathway (NarGHI, K00370, K00371, K00374), suggesting their inability to perform nitrate-reduction coupled P uptake. Their ability to denitrify on nitrite may be granted owing to the complete nitrite denitrifying pathway encoded in their genomes. Among all *Ca*. Accumulibacter, clade IIC members SSA1, SCUT-1, SCELCE-2, and SCELSE-8 lack the entire set of genes in the denitrification pathway; all these genomes were recovered from our lab-scale EBPR systems [20, 44, 45], representing to-date known non-denitrifying PAOs in the *Ca*. Accumulibacter lineage. Their inability to denitrify renders them an advantage to take up carbon sources free of the influence/inhibition from nitrate/nitrite under anoxic conditions [84], since they could recognize anoxic conditions as pseudo anaerobic as indicated by Cokro et al. [85]. In the remaining 38 genomes, except for *Ca*. Accumulibacter torridus SCELSE-9 and SCELSE-10 (IIK) and *Ca*. Accumulibacter SBRS (IIC), all other genomes encoded the first module for denitrification (nitrate reduction to nitrite). All clade IIB, IA, IG, IIA, IIF, and II-I members encoded nitrate reductase genes (*nap*AB). Clade IIC (except for SSA1, SCUT-1, SCELCE-2, and SCELSE-8 mentioned above), IIG, and IIH members encoded the respiratory nitrate reductase genes (*nar*GHI), suggesting their ability to perform denitrifying P removal on nitrate. The nitrate reduction pathway in clade IID and IC members occurred inconsistently; BATAC285 (IID) and ACC012 (IC) encoded the *nap*AB. UW12 (IID) and UWLDOIC (IC) encoded the *nar*GHI. Based on a phylogenetic analysis, both *nap*AB and *nap*GHI in *Ca*. Accumulibacter members are homologous. *nap*AB were ancestral genes inherited from the Rhodocyclaceae family. *nap*GHI were derived via HGT at the *Ca*. Accumulibacter LCA. The absence of *nap*AB or *nap*GHI genes in certain genomes suggest gene loss during evolution. In the second module of denitrification (nitrite reduction to nitric oxide), except for clade IIC members SSA1, SCUT-1, SCELCE-2, and SCELSE-8, all *Ca*. Accumulibacter genomes encode the cytochrome cd1-containing nitrite reductase gene (*nir*S), showing a highly conserved ability of *Ca*. Accumulibacter in nitrite reduction. Denitrifying bacteria carrying *nir*S was suggested to prefer high-carbon, low-oxygen environments and are widely distributed in aquatic systems, while the copper-containing nitrite reductase gene (*nir*K, K00368) encoding populations are typically located in areas with greater oxygen fluctuations [86]. This may indicate the original environment in which *Ca*. Accumulibacter acquired the *nir*S (a derived gene at their LCA). The third module (nitric oxide reduction to nitrous oxide) was only found in the unknown clade members (66-26, DS2011 and SCN18) and clade IIF members SCELSE-3, SCELSE-4, and SCELSE-6. Clades IC, IA, IG, IIA, IIF, and II-I members encoded the complete fourth module (nitrous oxide reduction to nitrogen). Previous research suggested that *Ca*. Accumulibacter clade I may collaborate with *Ca*. Competibacter to achieve complete denitrification [87]. Although only three *Ca*. Accumulibacter members (SCELSE-3, SCELSE-4, and SCELSE-6) have a complete denitrification pathway, all required blocks occurred in the pan *Ca*. Accumulibacter genome, implying that complete denitrification may also be achieved via the cooperation of different *Ca*. Accumulibacter clade members, contributing to the observed complete denitrification performance in *Ca*. Accumulibacter-dominated systems.

#### Heat shock protein

In the context of global warming, an improved understanding of the capability of different *Ca*. Accumulibacter members in surviving and resisting elevated temperature benefits extended applications of the EBPR process [88]. Previous studies suggested that at temperatures above 25°C, the carbon uptake rates of *Ca*. Competibacter (a group of GAO) surpasses that of *Ca*. Accumulibacter, resulting in unmatchable competitiveness of Ca. Accumulibacter and thus deteriorated EBPR performances [89–91]. Additionally, the anaerobic maintenance coefficients significantly increased for *Ca*. Accumulibacter, implying that they might be cryogenic microbes having difficulties surviving high temperatures, whereas stable long-term EBPR was demonstrated in a lab-scale reactor at 28°C. Clade IIF was found as the only *Ca*. Accumulibacter clade [33]. At 30°C, in lab-scale multi-cycle strategy aided EBPR systems, Shen et al. found that clade IIC members were highly abundant [34]. Coincidentally, the high-quality *Ca*. Accumulibacter genomes SCELSE-1, SSB1, and SSA1, which were recovered from high-temperature reactors, also belong to clade IIF or IIC [32, 33]. These results implied that different clades may differ in their ability to withstand high temperatures. Heat shock proteins (HSPs) are highly conserved molecular chaperones that are activated under stressful conditions, such as pH changes and high temperatures, and play a protective role against extreme environments [92]. The differences in their HSP encoding genes might result in different responses toward high temperatures by different *Ca*. Accumulibacter members. HSPs are divided into several families (including Hsp100s, Hsp90s, Hsp70s, Hsp60s, and Hsp20s) according to molecular weights and sequence homology [93]. Compared with other Hsp family members, Hsp20 (K13993) requires no activator, playing an important role in the heat resistances of microorganisms [93]. Li et al. [94] found that the transcription of Hsp20 mRNA markedly increased when *Sulfolobus solfataricus* P2 was subject to temperatures away from their optimal growth temperature, protecting the bacteria from heat stress. There are nine Hsp20 homologous gene families in all *Ca*. Accumulibacter genomes (Supplementary Spreedsheet S1). Phylogenetic analysis revealed that Hsp20 genes may have a multilineage origin and a complex evolutionary pattern. For example, OG0008859 was obtained from the Rhodocyclales family, which is present only in unknown clade members. Hsp20-1 (OG0002586) was derived via HGT from Gramaproteobacteria which occurred in clade IIC members (except SCUT-1), IIF, IIH, and IIK members, but was absent in clade IA, IC, IG, IIA, IIB, IID, IIG, and II-I members. Among all *Ca*. Accumulibacter genomes, 13 genomes were recovered from our EBPR systems operated at high temperatures; all of them encode the *hsp*20-1 gene. Additionally, six additional genomes encode the *hsp*20-1 gene, among which only one was recovered at 20°C (i.e. SK-01). The remaining genomes were all recovered from reactors operated at temperatures above 23°C (except Bin19 and UBA6585, for which the operating conditions of their resident reactors were not clear). In contrast, no *hsp*20-1 gene was observed in genomes recovered at 20°C or below (except SK-01, as mentioned above). A metatranscriptomic analysis was performed on an enrichment culture of clade IIC member SCUT-2. Results suggested that the *hsp*20-1 gene was highly transcribed (423 TPM) in the anaerobic phase (Fig. 6), while the transcription of other Hsp20 genes was low (the highest was 118 TPM). Similarly, high-level transcriptions of *hsp*20-1 were also observed in the SCUT-3 metatranscriptome during an EBPR cycle (up to 1840 RPKM) [95]. These results suggested that Hsp20s with low sequence similarity may have different functions in *Ca.* Accumulibacter, resulting in distinct heat resistance of different clade members. Hsp20-1 appears to be crucial for the survival and proliferation of *Ca*. Accumulibacter at high temperatures. Hsp90s is an HSP that requires activators [96]. These proteins are distinctly different in different clades. Two families of activating proteins (OG0003041 and OG0015504) (namely Activator-1 and Activator-2) are detected in the pan Accumulibacter genome. Activator-1 occurred in clade IA, IC, IG, IIA, II-I, and some clade IIF members (SCELSE-1 and SSB1). Activator-2 occurred only in clade IIH. Hsp90 and its activator were shown to play a significant role in stress tolerance and virulence in *Synechococcus*, *E. coli,* and *Pseudomonas aeruginosa* [97–100]. However, the absence of the activator in genomes recovered at high temperatures suggests that Hsp90 was not a key protein rendering these clade members high-temperature-tolerant. The remaining HSPs, such as Hsp15, Hsp30, etc., were highly conserved in different clades of *Ca*. Accumulibacter, which may not result in distinct heat resistance characteristics of different members.

### Implications

Genomes recovered at high temperatures significantly supplemented the existing *Ca*. Accumulibacter genome and gene databases, enhancing the understanding of their phylogeny, their carbon, nitrogen and phosphorus metabolisms, and their temperature adaptation mechanisms. The ancestral *ppk*1 was commonly used for clade classification, whereas inconsistencies were occasionally observed between *ppk*1 and single-copy core gene phylogenies, and/or ANI [32], such as *Ca*. Accumulibacter aalborgensis [33] and the two clade IIK genomes (*Ca*. Accumulibacter torridus SCELSE-9 and SCELSE-10) in this study. In this case, machine learning based on the whole-genome characteristics provides effective arbitrations for ambiguous clade identification (Fig. 1), rendering its promise to be considered in the future for robust clade identifications. Additionally, feature genes were identified for each clade. A majority of them were shown to be acquired via HGT, implying the role of HGT in shaping different clades. As a laterally derived gene which was considered to have played a key role in the emergence of the polyphosphate-accumulating trait of *Ca.* Accumulibacter [38], *ppk*2-1 seems to show increased resolution in differentiating different clades with higher intra-clade conservativity (Fig. 3), implying a special standing of the *ppk*2 gene. Compared with *ppk*1, the function of *ppk*2-1 is largely understudied. Future research may be carried out to understand the potential roles of these two polyphosphate kinase genes in the lineage evolution and speciation, the polyphosphate metabolic kinetics and inter-clade competitions.

Carbon sources are keys to EBPR. As two mainstay groups of PAOs in EBPR systems, *Ca*. Accumulibacter and *Tetrasphaera* are considered to occupy different ecological niches owing to their distinct carbon source preferences (VFAs vs. amino acids and sugars). Via pangenomic analysis, it seems that dicarboxylic amino acid utilization ability was highly conserved in the *Ca*. Accumulibacter lineage, suggesting inevitable competition between these two major groups of PAOs. For wastewaters containing high levels of dicarboxylic amino acids, the competition and its potential impacts on EBPR stability deserve attention [25]. The glucose metabolic potential is indeed scarce within the *Ca*. Accumulibacter lineage (Fig. 5), agreeing with the rarely observed glucose utilization capabilities of *Ca*. Accumulibacter consortia [26], whereas all *Ca*. Accumulibacter seems to be at the edge of using glucose. The acquisition of a *pst*G gene, such as the clade IIA member AALB and the clade IIG member BAT3C720, would be sufficient to confer them a complete glucose metabolic ability. Since glucose is a commonly used external carbon source in WWTPs, it would be interesting to know if this anthropogenic selection pressure/drive force is under way to transform *Ca*. Accumulibacter members into glucose users.

A seamless coupling of nitrate/nitrite reduction to phosphate uptake by PAOs is an ultimate goal to be pursued by WWTPs, especially for those which are short of influent organic carbon sources to support both nitrogen and phosphorus removal. Previous research suggested that no *Ca.* Accumulibacter member encoded a complete denitrifying pathway [9, 12]. The genomes recovered at high temperatures in this work provide examples for *Ca*. Accumulibacter which encode a complete denitrification pathway (SCELSE-3, SCELSE-4, and SCELSE-6, Fig. 5), and those which are completely unable to denitrify (SSA1, SCUT-1, SCELCE-2, and SCELSE-8, Fig. 5). Both groups of *Ca*. Accumulibacter are desirable for EBPR, since the former suggested that *Ca*. Accumulibacter alone could reduce nitrate/nitrite to nitrogen gas; and the latter would be valuable for WWTPs which do not have a well-defined anaerobic zone [81]. Additionally, all required genes for denitrification occurred in the pan *Ca*. Accumulibacter genome, implying the feasibility of relying solely on *Ca*. Accumulibacter for denitrifying phosphorus removal via the cooperation of different *Ca*. Accumulibacter members, avoiding the need for the involvement of additional denitrifying members (such GAOs), contributing to minimized carbon source demand for coupled nitrogen and phosphorus removal in WWTPs.

The occurrence pattern of the heat-shock protein *hsp*20-1 gene in *Ca*. Accumulibacter members (i.e. occurred in all high-temperature genomes but absent in almost all low-temperature genomes, Fig. 4) and its active transcription during the EBPR cycles (Fig. 6) suggested a critical role of this gene in the survival of *Ca*. Accumulibacter at elevated temperatures, whereas, this gene seems to have set up a clear boundary between low- and high-temperature members, where not only low-temperature residents are incompetent to survive high-temperature conditions, but the high-temperature survivors might neither be competitive with their low-temperature relatives at lower temperatures, which explains the inconsistent observations regarding the feasibility of *Ca*. Accumulibacter-mediated EBPR at high temperatures [28–30,87,88], and has important implications for successful operation of EBPR at high and/or changeable temperature conditions. This potential adaptability-isolation on the competition and cooperation of different *Ca*. Accumulibacter members and the resultant impacts on EBPR deserve further investigation.

## Conclusion

A total of 10 high-quality *Ca*. Accumulibacter genomes were recovered from high-temperature EBPR systems, including two clade IIH (*Ca*. Accumulibacter tropicus) and two clade IIK (*Ca*. Accumulibacter torridus) members, providing new data for the understanding of Ca. Accumulibacter. The metabolism required for the PAO phenotype is conserved in all *Ca*. Accumulibacter clades, whereas there are major differences in carbon metabolism and nitrogen metabolism pathways. All members are potentially dicarboxylic amino acid users, whereas only *Ca*. Accumulibacter aalborgensis AALB and *Ca*. Accumulibacter affinis BAT3C720 seemed capable of using glucose for EBPR. Clade IIF members SCELSE-3, SCELSE-4, and SCELSE-6 represented the to-date known genomes encoding a complete denitrification pathway, whereas IIC members SSA1, SCUT-1, SCELCE-2, and SCELSE-8 lack the entire set of genes in the denitrification pathway, representing to-date known non-denitrifying *Ca*. Accumulibacter. An HSP Hsp20-1 encoding gene was found to occur only in the genomes recovered at high temperatures, which may be responsible for the ability of clade IIF, IIC, IIH, and IIK members to survive high temperatures. Machine learning was used to identify the differences in genomic structure between clades, and to predict and validate the clade classification of different genomes. *ppk*2-base phylogeny showed improved resolution to characterize and differentiate different clades. Overall, the newly recovered *Ca*. Accumulibacter genomes at high temperatures provided significantly updated knowledge of the *Ca*. Accumulibacter lineage.

## Supplementary Material

Spreadsheet1_ycae049

Spreedsheet2_ycae049

Supporting_Material_ycae049

## Data Availability

All data generated or analyzed during this study are included in this published article. Metagenomic raw reads and MAGs were submitted to NCBI under BioProject No. PRJNA807832 and PRJNA771771. Metatranscriptomic data were submitted to NCBI under No. PRJNA807832. Other data were documented in the Supplementary Materials.
