# Supplementary material for: Two new clades recovered at high temperatures provide novel phylogenetic and genomic insights into Candidatus Accumulibacter
Source: ISME Commun. 2024 Apr 18;4(1):ycae049. doi: 10.1093/ismeco/ycae049 (PMC11131965; doi:10.1093/ismeco/ycae049)
Supplement: Supporting_Material_ycae049 [file supporting_material_ycae049.docx]

# Two new clades recovered at high temperatures provide novel phylogenetic and genomic insights into *Ca.* Accumulibacter

Xiaojing Xie^1,#^, Xuhan Deng^1,#^, Jinling Chen^1^, Liping Chen^1^, Jing Yuan^1^, Hang Chen^1^, Chaohai Wei ^1^^,3^, Xianghui Liu^2^, Guanglei Qiu^1,2,3,4*^

^1^ *School of Environment and Energy, South China University of Technology, Guangzhou 510006, China.*

^2^*Singapore Centre for Environmental Life Sciences Engineering, Nanyang Technological University, Singapore 637551, Singapore.*

^3^ *Guangdong Provincial Key Laboratory of Solid Wastes Pollution Control and Recycling, Guangzhou 510006, China*

^4^ *The Key Lab of Pollution Control and Ecosystem Restoration in Industry Clusters, Ministry of Education, Guangzhou 510006, China*

^*^ Corresponding Author: [qiugl@scut.edu.cn](mailto:qiugl@scut.edu.cn) (G.Q.)

^#^ Authors contributed equally towards this study

**Supporting Material:** 6 pages, 2 Spreadsheet

# *S1 Operation of the SBRs NTU30 and NTU35*

Two SBRs (operated in parallel at 30 ℃ (NTU30) and 35 ℃ (NTU35)) with a working volume of 1.59 L each were inoculated with activated sludge from a WWTP in Singapore. The SBRs were operated with 6 h cycles, including a 60 min feeding, a 20 min anaerobic, a 180 min aerobic, and a 100 min settling/decant stage. In each cycle, 0.74 L of synthetic wastewater (containing 193.8 mg/L acetate, 17.2 mg/L propionate and 8.8 mg/L PO4^3-^-P) was introduced into the reactor, with a resultant TOC/P molar ratio of 25:1. Temperature control was achieved using a proportional-integral-derivative temperature controller connected to heating jackets wrapping the reactors. The HRT and SRT in both reactors were 12.9 h and 25 d, respectively. The pH was automatically controlled at 6.80–7.50 by using a M200 transmitter connected to an acid/base (0.5 M HCl/NaOH) dosing system. The DO was maintained at 0.8–1.2 mg/L during the aerobic phase by using the same transmitter connected to a solenoid valve in the aeration system. During Days 175-220, sodium acetate and propionic acid concentrations in the feed for NTU35 were increased 1.6 times (resultant TOC/P molar ratio of 40:1), which was reduced to 1.2 times (TOC/P molar ratio of 30:1) from Day 220 onwards until the end of the experiment.

# *S2 Operation of the sequential batch reactor (SBR) SCUT*

The SBR SCUT with a working volume of 4.5 L was inoculated with activated sludge collected from a wastewater treatment plant (WWTP) in Guangzhou, China. The SBR was operated with 6 h cycles, including a slow-feeding phase (60 min), an anaerobic phase (20 min), an aerobic phase (180 min), and a settling/decant phase (100 min). Acetate was used as sole carbon source. In each cycle, 2.5 L of synthetic wastewater (containing 100 mg/L acetate, and 20 mg/L PO_4_^3-^-P) was fed into the reactor, with a resultant TOC/P molar ratio of 15:1. The hydraulic retention time (HRT) and sludge retention time (SRT) were 12 h and 15 d, respectively. The pH was automatically controlled at 7.0-7.5 by using a M200 transmitter (Mettler-Toledo, Switzerland) connected to an acid/base (0.5 M HCl/NaOH) dosing system. The dissolved oxygen (DO) was maintained at 0.8-1.2 mg/L during the aerobic phase by using the same transmitter connected to a solenoid valve in the aeration system. Temperature was controlled at 25℃ using a temperature controller connected to thermostatic water bath.

# *S3 DNA extraction and metagenomic analysis*

For SBRs NTU30 and NTU35, activated sludge samples were collected on Day 14, Day 56, Day 91, Day201, Day 280 and Day 301 from each SBR. Genomic DNA were extracted using the Fast DNATM 2 mL SPIN Kit for Soil samples (MP Biomedicals, CA, USA) following the manufacturer’s instructions, and stored at -80 °C prior to metagenomic analysis. Sequencing library preparation was performed using a modified version of the Illumina TruSeq DNA Sample Preparation protocol: 1 μg DNA was sheared on a Covaris S220 to approximately 300 bp, following the manufacturer’s recommendation. Size selection was performed on a Sage Science Pippin Prep instrument, using a 2% EtBr agarose cassette and selecting for a tight peak around 400 bp. Each library was tagged with a TruSeq LT DNA barcode (Illumina, CA, USA) to allow for library pooling prior to sequencing. Library quantitation was performed using the Picogreen assay (Invitrogen, CA, USA) and the average library size was determined by running the libraries on a Bioanalyzer DNA 7500 chip (Agilent, CA, USA). Library concentrations were normalized to 4 nM and validated by qPCR on a ViiA-7 realtime thermocycler (Applied Biosystems, CA, US), using qPCR primers recommended by Illumina in their qPCR protocol, and the Illumina PhiX control library was used as a standard. Libraries were then combined in one pool, which was sequenced across two lanes of an Illumina HiSeq2500 sequencing run at a read-length of 250 bp paired-end. Raw reads have been submitted to NCBI and are accessible under the BioProject No. PRJNA807832.

For the SBR SCUT, activated sludge was collected on Day 783. Genomic DNA were extracted using the OMEGA Soil DNA Kit (D5625-01) (Omega Bio-Tek, GA, USA), following the manufacturer’s instructions, and stored at -80 °C prior to further analysis. The quantity and quality of the extracted DNA were measured using a Qubit™ 4 Fluorometer (with WiFi: Q33238; Qubit™ Assay Tubes: Q32856; Qubit™ 1X dsDNA HS Assay Kit: Q33231) and 1% agarose gel electrophoresis, respectively. Genomic library was constructed following the Illumina TruSeq DNA Sample Preparation Guide. Illumina NovaSeq sequencing run at a read-length of 150 bp paired-end. Low-quality reads and adapter sequences were removed using fastp 0.20.1 (1). metaSPAdes v.3.13.0 was used to assemble high-quality reads to contigs (2). The contigs were binned into metagenome-assembled genomes (MAGs) using MetaBAT2 v1.7 and CONCOCT v1.1 (3, 4). CheckM v1.0.18 was used to evaluate the completeness and contamination of MAGs (5). The taxonomic classifications of obtained MAGs were performed using the Genome Taxonomy Database Toolkit (GTDB-Tk v. 2.3.0) (6). MAGs with completeness below 95% and contamination over 6% were discarded. BBMap v38.96 was used to calculate the relative abundance of each *Ca*. Accumulibacter MAGs (7). All MAGs were annotated using RAST annotation server (8). Except for quality control by fastp, annotation by RAST and relative abundance calculation by BBMap and taxonomic classifications for obtained MAGs by the GTDB-TK, all analyses, including metaSPAdes, MetaBAT2, COCOCT, CheckM2, were performed on the KBase platform (9). Raw reads have been submitted to the National Center for Biotechnology Information (NCBI) and are accessible under BioProject No. PRJNA807832.Raw reads have been submitted to NCBI and are accessible under the BioProject No. PRJNA771771.

# *S4 Full cycle study*

An anaerobic-aerobic full cycle study was performed on Day 783 in the SCUT reactor. The full cycle contains an 80 min anaerobic stage (including a 60-min slow-feeding stage and a 20-min anaerobic stage afterwards) and a 180 min stage. Water and activated sludge samples was collected at 5, 15, 30, 45, 60, 80, 105, 120, 150, 180, 210 and 260 min for TOC, PO_4_^3-^-P, PHA and glycogen analyses.

PO_4_^3-^-P concentrations were determined following the Standard Methods (APHA, 1999). Total organic carbon (TOC) was measured using a TOC analyzer (Shimadzu, Japan). PHA analyses were performed according to Oehmen et al. (2005), using a Trace Ultra Gas Chromatography (equipped with a DB-5MS column, 30 m ×0.25 mm, Agilent Technology, USA) coupled to a DSQ II mass spectrometer (Thermo Scientific, USA). Glycogen analyses were carried out according to the method described by Kristiansen et al. (2013). Lyophilized activated sludge was resuspended in 5 mL of 0.9 M HCl and digested at 100 ℃ for 5 h. Glucose equivalents in the supernatant were quantified using a HPLC (E2695, Waters, US) equipped with a HyREZ XP column (Dionex, Thermo Fisher, Denmark).

Activated sludge samples were also collected just before the start of the full cycle (0 min), at 5 min (anaerobic phase), 30 min (anaerobic phase), 105 min (anaerobic phase), and 120 min (aerobic phase), snap-frozen in liquid N_2_, and stored at -80 ^o^C before RNA extraction for metatranscriptomic analysis.

# *S5 Metatranscriptomic analysis*

Activated sludge samples were collected just before the start of a sequencing batch reactor (SBR) cycle (0 min), and at 5 min (anaerobic phase), 30 min (anaerobic phase), 105 min (aerobic phase), and 120 min (aerobic phase) of the SBR cycle. The samples were snap-frozen in liquid N_2_ and stored at −80 °C before the extraction of ribonucleic acid (RNA) for metatranscriptomic analysis. For metatranscriptomic analysis, 10 ng RNA was loaded for each sample. Total RNA was extracted using the RNA PowerSoil® Total RNA Isolation Kit (Omega Bio-Tek, GA, USA). The quality and quantity of the extracted RNA were measured using 1.5% agarose gel electrophoresis and UV spectrophotometer, respectively. cDNA library was constructed using a TruSeq Standard mRNA LT Sample Prep Kit (Illumina, CA, USA). The quality and quantity of the library were measured using Agilent Bioanalyzer and Promega QuantiFluor, respectively. Illumina NovaSeq sequencing run at a read-length of 150 bp paired-end.

Fastp (1) and SortMeRNA (10) were used to remove adaptation sequences and ribosomal ribonucleic acids (rRNAs). Filtered reads were mapped to the corresponding Ca. Accumulibacter draft genome (i.e., SCUT-2) using BBMap version 38.96 (7) and were normalized to transcript per million (TPM). Genes with TPM > 100 were considered to be highly transcribed. Raw reads and draft genomes obtained were submitted to NCBI under BioProject No. PRJNA807832 and No. PRJNA771771.

## Spreadsheet 1

Genomic information and metabolic pathways. Sheet-1 Genomic information includes clade classification, assembly accession, integrity, GC%, and genome size. Sheet-2 Pathway modules of each genome in nitrogen metabolism, central carbohydrate metabolism and carbon fixation. Sheet-3 Presence or absence of phosphorus removal-related genes in the representative genome. Sheet-4 Presence or absence of key genes in carbon metabolism and nitrogen metabolism in each genome. Sheet-5 Presence or absence of heat shock protein in each genome. Sheet-6-Sheet-18 KEGG metabolic annotation for the representative genomes.

## Spreadsheet 2

Information of machine learning. Sheet-1. Pan *Ca.* Accumulibacter gene clusters and the number of genes in each homologous gene cluster. Sheet-2. Pan *Ca.* Accumulibacter gene clusters and the number of genes in each clade. Sheet-3 Input features of the machine learning model after filtering out unimportant features with R. Sheet-4 The 20 most important features of LB model output. Sheet-5 Machine learning is used to analyze the input features of SCELSE-9 and SCEL-10 and to predict the results.

**Reference**

1. Chen S, Zhou Y, Chen Y, Gu J. Fastp: An Ultra-Fast All-in-One Fastq Preprocessor. Bioinformatics. 2018;34(17):i884-i90.

2. Nurk S, Meleshko D, Korobeynikov A, Pevzner PA. Metaspades: A New Versatile Metagenomic Assembler. Genome Research. 2017;27(5):824-34.

3. Kang DD, Li F, Kirton E, Thomas A, Egan R, An H, Wang Z. Metabat 2: An Adaptive Binning Algorithm for Robust and Efficient Genome Reconstruction from Metagenome Assemblies. PeerJ. 2019;7:e7359.

4. Alneberg J, Bjarnason BS, de Bruijn I, Schirmer M, Quick J, Ijaz UZ, et al. Binning Metagenomic Contigs by Coverage and Composition. Nature Methods. 2014;11(11):1144-6.

5. Parks DH, Imelfort M, Skennerton CT, Hugenholtz P, Tyson GW. Checkm: Assessing the Quality of Microbial Genomes Recovered from Isolates, Single Cells, and Metagenomes. Genome Research. 2015;25(7):1043-55.

6. Chaumeil PA, Mussig AJ, Hugenholtz P, Parks DH. Gtdb-Tk: A Toolkit to Classify Genomes with the Genome Taxonomy Database. Bioinformatics. 2019;36(6):1925-7.

7. Bushnell B, editor Bbmap: A Fast, Accurate, Splice-Aware Aligner2014.

8. Aziz RK, Bartels D, Best AA, DeJongh M, Disz T, Edwards RA, et al. The Rast Server: Rapid Annotations Using Subsystems Technology. BMC Genomics. 2008;9(1):75.

9. Arkin AP, Cottingham RW, Henry CS, Harris NL, Stevens RL, Maslov S, et al. Kbase: The United States Department of Energy Systems Biology Knowledgebase. Nature Biotechnology. 2018;36(7):566-9.

10. Kopylova E, Noé L, Touzet H. Sortmerna: Fast and Accurate Filtering of Ribosomal Rnas in Metatranscriptomic Data. Bioinformatics. 2012;28(24):3211-7.
